# Angiotensin II may be useful for the treatment of hypotension in distributive shock, but a safe and efficacious dose is unknown

**DOI:** 10.1186/cc13350

**Published:** 2014-03-17

**Authors:** LW Busse, E Brasha, DL Davison, MG Seneff, J Honig, ZK Alotaibi, LS Chawla

**Affiliations:** 1 Inova Fairfax Health System, Falls Church, VA, USA; 2 George Washington University, Washington, DC, USA; 3 Prince Sultan Medical Military City, Riyadh, Saudi Arabia

## Introduction

Patients with distributive shock who require high-dose vasopressors have a high mortality. Vasopressors belong to two classes: catecholamines and vasopressin analogs. Each class has limitations. Patients receiving catecholamines often develop tachyphylaxis and metabolic complications (for example, lactic acidosis). Vasopressin analogs can cause mesenteric or myocardial ischemia and oliguria. Angiotensin II (ATII) is an endogenous peptide that increases blood pressure and aldosterone production. Preclinical data suggest a role for ATII in the treatment of sepsis-associated acute kidney injury. ATII may prove useful in patients who remain hypotensive despite catecholamine and vasopressin therapy. This is the first randomized clinical trial to date which seeks to evaluate ATII for use in distributive shock.

## Methods

Twenty patients with distributive shock and a cardiovascular Sequential Organ Failure Assessment score ≥4 were randomized to either ATII infusion (*n *= 10) or placebo (*n *= 10) plus standard of care. ATII was started at a dose of 20 ng/kg/minute, and titrated per a protocolized schedule for a goal of maintaining a mean arterial pressure (MAP) of 65 mmHg. The infusion (either ATII or placebo) was continued for 6 hours and then titrated off. The primary endpoint was the effect of ATII on the standing dose of norepinephrine required to maintain a MAP of 65 mmHg. Secondary endpoints included the effect of ATII on urine output, serum lactate and creatinine clearance, as well as 30-day mortality.

## Results

The mean age was 62.9 ± 15.8 years. Of the patients, 75% were male, 45% were Caucasian and 40% were African American. The 30-day mortality for the two groups were similar for the ATII cohort and the placebo cohort (50% vs. 60%, *P *= 1.00). ATII resulted in marked reduction in norepinephrine dosing in all patients. The mean norepinephrine dose for the placebo cohort was 20.1 ± 16.8 μg/ minute vs. 7.3 ± 11.9 μg/minute for the ATII cohort (*P *= 0.022). The most common adverse event was hypertension, which occurred in 20% of patients receiving ATII.

## Conclusion

ATII is an effective vasopressor agent in patients with distributive shock requiring multiple vasopressors. The initial dose range of ATII that appears to be appropriate for patients with distributive shock is 2 to 10 ng/kg/minute. Further studies to assess the use of ATII in patients with distributive shock are warranted.

